# The role of admixture in the rare variant contribution to inflammatory bowel disease

**DOI:** 10.1186/s13073-023-01244-w

**Published:** 2023-11-15

**Authors:** Courtney Astore, Shivam Sharma, Sini Nagpal, NIDDK IBD Genetics Consortium, David J. Cutler, John D. Rioux, Judy H. Cho, Dermot P. B. McGovern, Steven R. Brant, Subra Kugathasan, I. King Jordan, Greg Gibson

**Affiliations:** 1 Center for Integrative Genomics and School of Biological Sciences, Georgia Institute of Technology, Krone EBB1 Building, 950 Atlantic Drive, Atlanta, GA 30332 USA; 2 Department of Human Genetics, Emory University School of Medicine, Atlanta, GA 30322 USA; 3 Department of Medicine, Université de Montréal and the Montreal Heart Institute Research Center, Montreal, QC H1Y3N1 Canada; 4 Charles Bronfman Institute for Personalized Medicine, Icahn School of Medicine at Mount Sinai, New York, NY 10029 USA; 5 Department of Medicine, Rutgers Robert Wood Johnson Medical School, New Brunswick, NJ 08901 USA; 6 Department of Genetics and the Human Genetics Institute of New Jersey, Rutgers University, Piscataway, NJ 08554 USA; 7 Meyerhoff Inflammatory Bowel Disease Center, Johns Hopkins University School of Medicine, Baltimore, 21287 USA; 8 Immunology Research Institute, Cedars-Sinai Medical Center, Los Angeles, CA 90048 USA; 9 Department of Pediatrics, Emory University School of Medicine, and Children’s Healthcare of Atlanta, Atlanta, GA 30322 USA

**Keywords:** Trans-ancestry, Crohn’s disease, *NOD2*, Genetic risk assessment, Whole genome sequencing

## Abstract

**Background:**

Identification of rare variants involved in complex, polygenic diseases like Crohn’s disease (CD) has accelerated with the introduction of whole exome/genome sequencing association studies. Rare variants can be used in both diagnostic and therapeutic assessments; however, since they are likely to be restricted to specific ancestry groups, their contributions to risk assessment need to be evaluated outside the discovery population. Prior studies implied that the three known rare variants in *NOD2* are absent in West African and Asian populations and only contribute in African Americans via admixture.

**Methods:**

Whole genome sequencing (WGS) data from 3418 African American individuals, 1774 inflammatory bowel disease (IBD) cases, and 1644 controls were used to assess odds ratios and allele frequencies (AF), as well as haplotype-specific ancestral origins of European-derived CD variants discovered in a large exome-wide association study. Local and global ancestry was performed to assess the contribution of admixture to IBD contrasting European and African American cohorts.

**Results:**

Twenty-five rare variants associated with CD in European discovery cohorts are typically five-fold lower frequency in African Americans. Correspondingly, where comparisons could be made, the rare variants were found to have a predicted four-fold reduced burden for IBD in African Americans, when compared to European individuals. Almost all of the rare CD European variants were found on European haplotypes in the African American cohort, implying that they contribute to disease risk in African Americans primarily due to recent admixture. In addition, proportion of European ancestry correlates the number of rare CD European variants each African American individual carry, as well as their polygenic risk of disease. Similar findings were observed for 23 mutations affecting 10 other common complex diseases for which the rare variants were discovered in European cohorts.

**Conclusions:**

European-derived Crohn’s disease rare variants are even more rare in African Americans and contribute to disease risk mainly due to admixture, which needs to be accounted for when performing cross-ancestry genetic assessments.

**Supplementary Information:**

The online version contains supplementary material available at 10.1186/s13073-023-01244-w.

## Background

Rare variants contribute modestly to the overall genetic burden of disease risk within a population, but can make an over-sized contribution to the susceptibility of some individuals who are carriers of ultrarare loss of function (LoF) variants [1, 2]. Previously, many researchers speculated that so-called goldilocks alleles, carried by one in ten to one in 50 people and with additive effects in the odds ratio range of 1.2 to 1.5, might explain so-called missing heritability [3, 4]. While rare variants in this effect size range and lower have been shown to account for some of the genetic variance, it is a minority contribution, recently estimated at less than 2% of the phenotypic variance across 22 common traits [1]. Nevertheless, because the effect sizes of rare variants are often greater than those of common variants that are more likely to have escaped purifying selection, it remains important to establish the rare variant contributions to disease and complex traits [5–7].

There are two major compelling concerns: (1) discovery and validation of causal effects and (2) establishment of whether or not they are consistent across ancestry groups [8, 9]. Regarding discovery, given 50,000 fully genotyped controls, a variant with a frequency of 0.02 and an odds ratio of 1.2 will generally require more than 30,000 whole exome-sequenced individuals to exceed the standard genome-wide significance threshold for association. Studies powered for consistent discovery at this level are just now becoming available. For example, Sazonovs et al. [10] meta-analyzed exome-wide associations studies from 35 cohorts in the International IBD-Genetics Consortium, which led to the identification of 45 rare coding variants associated with Crohn’s disease, of which 25 were inferred to be likely-causal. This analysis identified nine new genes involved in IBD pathology and implicated a role for mesenchymal cells in intestinal inflammation.

Differences in allele frequency, disease prevalence, sample size, and effect size all contribute to variability across populations in the replication of genetic associations [8]. For example, a study of 61,407 cases and almost a million controls found that just 20% of the 40 significant associations with lung cancer are consistent across European, East Asian, and African populations [11]. These issues are particularly prominent for rare variants for which the variance in allele frequencies is high relative to the risk (or in some cases, protective) minor allele. According to standard population genetic theory, the number of derived rare variants should be similar in both populations following a separation event, such as the Out-of-Africa migration that gave rise to modern European and Asian ancestries [12]. However, most rare variants are not shared across the major ancestry groups [13, 14]. African populations harbor greater nucleotide diversity and more rare variants at most loci, but markedly higher sampling in genetic studies to date has nevertheless resulted in greater discovery of associations in Europeans [15]. An early study of the impact of rare coding variants in *NOD2* for Crohn’s disease risk argued that most if not all the risk in African American due to two variants (G908R and 1007fs) was due to admixture since both are absent from West African genomes, and similarly, a third, R702W, is five-fold lower [16]. These variants are in linkage equilibrium with one another.

Although personalized genomic medicine has not yet reached clinical implementation for inflammatory bowel disease, many groups are working toward the objective of incorporating genetic and multi-omic assessment into therapeutic recommendations [17]. Recent evidence that rare variant-derived polygenic risk scores are likely to be more transferrable across continental ancestry groups than common variant PRS [18] and to more consistently discriminate the highest risk individuals, makes evaluation of rare variant contributions across ancestries imperative. Soon after the discovery that NOD2 makes an unusually large contribution to Crohn’s disease risk in Europeans, potential clinical utility in prediction of fibrostenotic disease progression or need for surgery was considered [19, 20]. Although they are not currently used for this purpose, NOD2 genotyping is often deployed as a covariate in basic research, and NOD2-driven disease may be responsive to gp130 blockade as a complement to anti-TNF therapy [21]. Population differences in the prevalence of such cases would create potential disparities in clinical outcomes.

Here we examine the contribution of the 25 Sazonovs et al. [10] rare CD variants to IBD in an African American whole genome sequencing (WGS) cohort comprising 1774 affected individuals and 1644 controls [22]. We validate a consistent reduction in allele frequency (AF) relative to Europeans with gnomAD [23] summary-level data and use chromosomal assignment of rare variant phasing to confirm that almost all the rare variant CD risk discovered in Europeans contributes to risk in African Americans due to admixture. Similarly, 45 rare variants discovered in the UK Biobank (UKB) and FinnGen across ten disease classes from Sun et al. [24] have prevalence in African Americans mostly explained by admixture. These results underline the pressing need for discovery-oriented whole exome and genome sequencing in very large cohorts of diverse ancestry if the role of rare variants in disease is to be better understood and used for equitable genetic analysis.

## Methods

### IBD risk genes and variants

Forty-five IBD risk variants from Sazonovs et al. [10] were extracted from their supplemental table 3. Variants with a status of “Known causal candidate”, “New variant in known locus”, or “New locus” were included in our analyses, since those 25 variants were argued to be causal for IBD. These 25 variants spanned 14 genes on 10 chromosomes and 6 were protective (OR_meta_ < 1) while 19 were risk (OR_meta_ > 1) minor as well as derived alleles in the predominantly European-ascertained cohorts.

### Allele frequencies of IBD risk variants across European and African cohorts

The allele frequencies (AF) of the IBD risk variants in European and African American IBD cases and control cohorts were assessed in multiple large population studies. Sazonovs et al. [10] reported the AF of the IBD risk variants for two batches of IBD cases and controls in their supplementary table 3. The TWIST batch consisted of 6109 IBD cases and 6064 controls, and the Nextera batch consisted of 11,125 IBD cases and 25,145 controls. Serving as additional controls, the AFs of the IBD risk variants for the Non-Finish European (*n* = 34,029) and African (*n* = 20,744) cohorts from gnomAD v3.1 WGS [18] were extracted using the Ensembl Variant Effect Predictor (VEP) tool [25]. In addition, the AFs for White British IBD cases (*n* = 5660) and controls (*n* = 375,309) were assessed from the UK BioBank 450K WES data using the --freq function in Plink 1.9 [26] as well as the –a2-allele function to ensure consistency of the ascertained allele. The AFs of IBD risk variants were similarly assessed in the IBD-GC African American IBD cases (*n* = 1774) and controls (*n* = 1644) from WGS data of Americans with African ancestry reported by the IBD-GC in [22], freely available by application to the IBD-GC Data Commons at https://www.ibdgc.org/data/ or via dbGaP dataset phs001642.v1.p1.

### EP-score variant annotations

EP-scores (evolutionary probabilities) for each of the Sazonovs et al. [10] variants were extracted from the EP-score matrix from [27], which has an EP-score for each of the 20 amino acid alleles for each position in the human proteome. Variants with EP-score less than 0.0022 are highly enriched for known pathogenic mutations.

### Association testing and variant carrier assessment

Fisher’s exact test was performed on the African American IBD and control cohorts using the --assoc function in Plink 1.9 [26], without adjusting for covariates since power was low. Carriers (homozygous alternate and heterozygous) and non-carriers (homozygous reference) were labeled for the African American cohort for each of the Sazonovs et al. variants present in the WGS data by recoding the data using --recode, --a2-allele, and –extract functions in Plink 1.9 and annotating the genotypes. This allowed us to assess the number of Sazonovs rare variants carried by each individual.

### Computation of variance explained and allelic burden

The amount of variance explained by each rare variant was computed as 2*p*(1-*p*)(lnOR)^2^ where *p* is the allele frequency and lnOR is the natural logarithm of the odds ratio between heterozygote carriers and major allele homozygotes reported in Sazonovs et al. [10] or computed by us in the IBD-GC WGS African American dataset [22]. In addition, we computed the excess of cases attributable to each rare variant carrier, disregarding homozygotes which will be extremely rare, as follows. Given the allele frequency in controls, and heuristically assuming 1% disease prevalence in all populations, we can compute the number of expected carriers per 100,000 people if there is no effect of the allele, which is just the number of heterozygotes 2*p*(1-*p*)×1000. We then increment this number to generate the observed odds ratio, computed as the product of heterozygous cases×homozygous controls divided by heterozygous controls×homozygous cases. For example, if the odds ratio for carriers of an allele with *p* = 0.01 is 1.3, then 25 heterozygous cases are expected (25×98030/1960×975 = 1.3), compared with 20 for no effect (20×98030/1960×980 = 1.0), resulting in an excess of 5 cases per 100,000 individuals.

### Phasing and ancestry inference

A total of 139,550,111 variants on 22 autosomes were extracted from the 3418 African American WGS samples [22]. The ancestry phase of each allele in this extracted dataset was corrected by matching them to variants in phase 3 WGS data from 1000 Genomes Project [28]. Subsequently, a dataset containing 40,784,703 biallelic SNPs was phased using Beagle v5.2 [29] with imputation mode turned off and window size reduced to 10 because of the dense nature of WGS data.

Continental and local ancestry inference was carried out using Admixture v1.3 [30] and Gnomix [26], respectively; 404 European (CEU, GBR, IBS, and TSI) and 405 African (ESN, GWD, MSL, and YRI) individuals from 1000 Genomes Project were used as reference samples for both analyses [28]. For continental ancestry inference, the variants were pruned for linkage disequilibrium (window size: 100; step size: 10; and *r*^2^: 0.025) and minor allele frequency (cutoff: 1%) using plink v1.9 [26] to yield the final dataset containing 251,042 variants. For local ancestry inference, Gnomix [31] was used in training the model for up to 12 generations, and the window size was dropped to 0.1 to adjust for WGS sequencing data. Chromosome painting for one case and one control individual who are carriers for 4 of the inferred causal Sazonovs et al. variants was generated using Tagore (https://github.com/jordanlab/tagore).

### European- and African-derived PRS

Polygenic risk scores were computed based on liability-scale weights at 215 known IBD risk variants reported in the latest IBD GWAS- release [32], estimated from the UK BioBank and the IBD-GC WGS African American (AA) cohorts as described in [22]. The scores were generated using plink v1.9 [26] as described in [22].

### Nucleotide diversity of the CDS regions of IBD genes

An adjusted average nucleotide diversity metric was computed for each gene’s coding sequence (CDS) region from the African American and white British European IBD case and control cohorts. The CDS region of each gene was identified using the latest GRCh38 *Homo sapiens* RefSeq genomic annotation file downloaded from https://ftp.ncbi.nlm.nih.gov/refseq/H_sapiens/annotation/GRCh38_latest/refseq_identifiers/GRCh38_latest_genomic.gff.gz. African American IBD case and control cohorts were created for each locus, only including individuals who were inferred to be homozygous African-derived for the given haplotype. To reduce risk of ascertainment bias, we randomly down-sampled ten UKBB white British European IBD case and control cohorts to match the sample size of the African American cohort for assessing nucleotide diversity. The vcftools --site-pi function was used to assess the nucleotide diversity at each position [33]. The adjusted average nucleotide diversity adjusts for total length of the CDS region of the gene as well as the number of CDS region sites present in the sequencing data for the respective cohort.

### Gene annotations

The predicted loss of function intolerance (pLI) score [34], loss-of-function observed/expected upper bound fraction (LOEUF) score [35], and residual variation intolerance score (RVIS) metrics were obtained for each of the Sazonovs et al. [10] genes. The pLI score, LOEUF score, as well LoF obs/exp ratio, were obtained from gnomAD using the bigBedToBed tool to facilitate extraction (https://hgdownload.soe.ucsc.edu/gbdb/hg38/gnomAD/pLI/pliByGene.bb). RVIS scores and percentiles were extracted for each gene with AF threshold of 0.1% for African American and European Americans from (https://genic-intolerance.org/data/GenicIntolerance_v3_12Mar16.txt).

### Rare variants in other disease groups

Nine hundred seventy-five unique, significant disease-variant associations were determined via the meta-analysis of exome-wide association study summary results for UKBB and FinnGen from Sun et al. [24] supplementary materials. Disease group cluster mappings were extracted from their supplemental table 2 and mapped to their supplemental table 3 diseases. These associations spanned 16 disease groups, 204 UKBB diseases, 216 FinnGen diseases, 534 variants, 21 chromosomes, and 11 variant consequences. Some diseases map to multiple disease groups. There were 82 disease-variant associations with a MAF_UKBB_ < 0.01, 71 disease-variant associations with a MAF_FinnGen_ < 0.01, and 96 disease-variant associations with a MAF_UKBB_ or MAF_FinnGen_ < 0.01. VEP was used to assess the gnomAD NFE (non-Finnish European) and AFR AFs for the 534 unique variants. Ultimately, we identified 45 unique variants with a gnomAD NFE AF < 0.01. These 45 variants corresponded to 84 significant disease-variant associations and 13 unique disease groups. Ages in units of generations were downloaded from the joint clock (mutation and recombination clock) mode computation for the Thousand Genome Project (TGP) cohort. Fifteen of the rare variants were not present in the Human Genome Dating database [36] at https://human.genome.dating/. One variant, 15:48134287:A:G, was the minor allele in Europeans (UKBB, FinnGen, and gnomAD) but the major allele in Africans, so was excluded from the analysis. This left 39 of the 45 variants that were present in the African American IBD/control cohort, of which 23 variants are on the 13 chromosomes phased with local ancestry performed in our IBD analysis. The proportion of African alleles and proportion of European alleles were assessed for the haplotypes corresponding to the 23 variants.

## Results

### Rare causal variants for Crohn’s disease are even more rare in African Americans

**Fig. 1:**
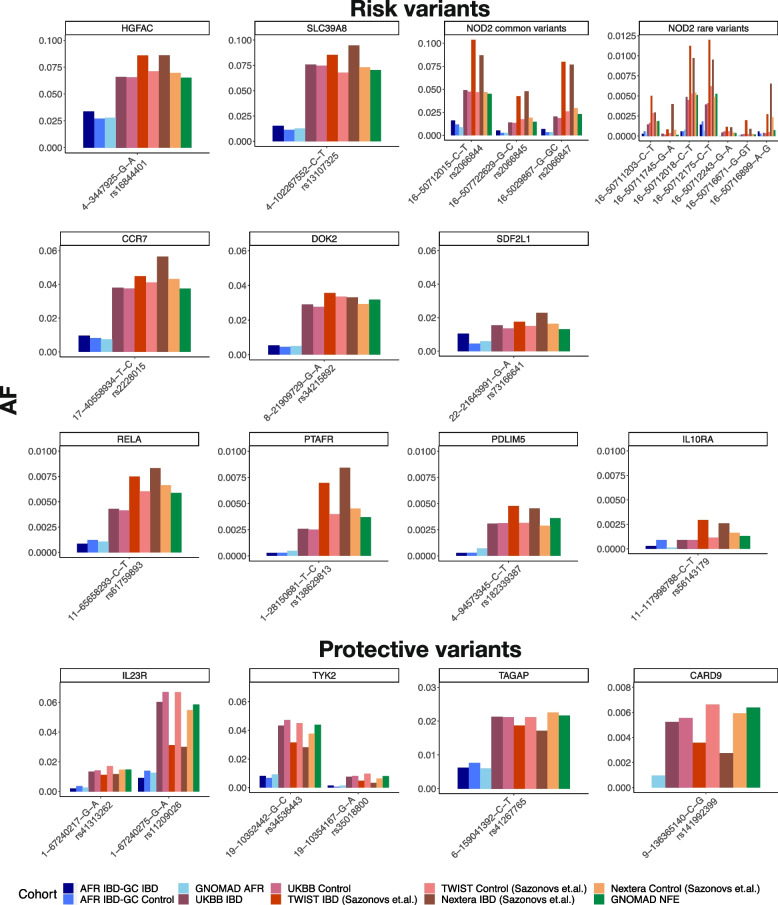
Minor allele frequencies of rare variants across European and African cohorts. Bars show MAF of the 25 rare variants reported to be causal for IBD by Sazonovs et al. [10], grouped by risk or protective status, with colors representing the cohorts shown in the legend at bottom. The SNP ID coordinates are shown for GRch38 with corresponding rsIDs from dbSNP; rs IDs for the ultra-rare *NOD2* SNPs are from left to right the same order as top to bottom in Table 1

Similar to previous results at the *NOD2* locus [16], across all 11 Sazonovs et al. [10] risk genes, and 4 protective genes, rare variants inferred to be causally related to Crohn’s disease in European ancestry (EA) individuals are one-fifth as prevalent in African Americans (AA). All comparisons were significant by Fisher’s exact test. Figure 1 shows this graphically for all 25 variants, with data summarized in Table 1. Reduced minor allele frequency (MAF) was observed consistently for two common risk variants in EA with MAF > 0.05 at *HGFAC* and *SLC30A8*, for six rare risk variants with 0.01 < MAF < 0.05 at *CCR7*, *DOK2*, *SDF2L1*, and *NOD2* (all four are well-established coding mutations), and a total of 11 very rare or ultra-rare risk variants with MAF < 0.01, one each at *RELA*, *PTAFH*, *PDLIM5*, *IL10RA*, and seven at *NOD2*. Concordantly, six rare variants in *IL23R*, *TYK2*, *TAGAP*, and *CARD9* that are protective in EA were even more rare in AA. In all cases, the difference was replicated in two AA cohorts (our IBD-GC case-control WGS cohort and gnomAD AA) and three EA datasets (UK Biobank, Sazonovs et al., and gnomAD Non-Finnish Europeans).

**Table 1:** Allele frequencies and effects of rare coding variants affecting CD susceptibility

**Gene**	rsID	**Variant**	**AA MAF**^**d**^	**EU MAF**^**d**^	**Ratio**^**e**^	**AA OR**^**f**^	**EU OR**^**g**^	**AA%VE**	**EU%VE**	**AA xs**	**EU xs**
**Rare variants associated with risk**											
HGFAC	rs16844401	R516H	0.028	0.070	0.40	1.25	1.17	0.27	0.32	13	19
SLC93A8	rs13107325	A391T	0.013	0.070	0.19	1.36	1.21	0.24	0.47	9	29
*CCR7*	rs2228015^a^	M7V	0.007	0.038	0.18	1.17	1.15	0.03	0.14	2	10
*DOK2*	rs34215892^a^	P274L	0.005	0.032	0.16	1.18	1.25	0.03	0.31	2	14
*SDF2L1*	rs73166641^a^	R161H	0.006	0.013	0.46	2.30	1.24	0.83	0.12	15	7
*NOD2*	rs2066844^a^	R702W	0.009	0.045	0.2	1.36	2.07	0.17	4.55	6	77
*NOD2*	rs2066845^a^	G908R	0.003	0.015	0.2	1.96	2.34	0.27	2.14	6	36
*NOD2*	rs2066847^a^	fs1007	0.003	0.023	0.13	2.03	3.00	0.30	5.42	6	79
*NOD2*	rs104895431^b^	S431L	0.0004	0.0019	0.21	0.46	1.74	0.05	0.12	0	3
*NOD2*	rs104895438^c^	A612T	0.0000	0.0002	n/a	n/a	3.12	n/a	0.05	0	1
*NOD2*	rs5743277^b^	R703C	0.0008	0.0051	0.16	0.93	1.88	0.0008	0.40	1	9
*NOD2*	rs61747625^b^	A755V	0.0007	0.0053	0.13	0.77	2.34	0.01	0.76	2	14
*NOD2*	rs104895443^c^	E778K	0.0000	0.0004	n/a	n/a	3.03	n/a	0.10	0	2
*NOD2*	rs373550987^c^	splice	0.0000	0.00006	n/a	n/a	4.97	n/a	0.03	0	1
*NOD2*	rs104895467^c^	N852S	0.0001	0.0007	0.14	1.85	2.31	0.008	0.10	0	1
*RELA*	rs61759893^b^	D291N	0.0011	0.006	0.18	0.69	1.46	0.03	0.17	1	5
*PTAFR*	rs138629813^b^	N114S	0.0005	0.0038	0.13	0.93	1.70	0.0005	0.21	1	5
*PDLIM5*	rs182339387^b^	splice	0.0007	0.004	0.18	0.93	1.61	0.0007	0.18	1	5
*IL10RA*	rs56143179^b^	P295L	0.0001	0.0013	0.08	0.31	2.11	0.03	0.14	0	3
**Rare variants associated with protection**											
*IL23R*	rs41313262^a^	V362I	0.003	0.015	0.2	0.54	0.70	0.23	0.38	3	8
*IL23R*	rs11209026	R381Q	0.013	0.07	0.19	0.64	0.49	0.51	6.63	9	61
*TYK2*	rs35018800^b^	A928V	0.001	0.008	0.13	2.32	0.60	0.14	0.41	1	6
*TYK2*	rs34536443^a^	P1104A	0.009	0.044	0.21	1.22	0.72	0.07	0.91	0	21
*TAGAP*	rs41267765^a^	E147K	0.006	0.022	0.27	0.81	0.79	0.05	0.24	2	9
*CARD9*	rs141992399^b^	splice	0.001	0.006	0.17	n/a	0.47	n/a	0.68	0	6

Last two columns show the expected excess (xs) of cases per 100,000 individuals assuming prevalence 1%

*VE* Variance explained (VE)

^a^Rare variant (0.01 < EU MAF < 0.05); ^b^Very rare variant (0.001 < EU MAF < 0.01); ^c^Ultra-rare variant (EU MAF ≤ 0.001)

^d^MAF in this table refers to gnomAD African or Non-Finnish European frequencies

^e^Ratio of African American to European allele frequency in gnomAD

^f^Odds ratios from AA-WGS cohort; not computed if MAF < 0.001

^g^Odds ratios reported from Sazonovs meta-analysis

As shown in Table 2, we also identified 4 variants in these loci with MAF > 0.01 in AA which have low evolutionary probability, namely EP-scores in the range of those of known pathogenic variants, but are nearly absent in Europeans: one in *SDF2L1*, two in *PTAFR*, and one in *IL10RA*. The latter variant may be protective since the minor allele frequency is 4.5% in both the AA-WGS controls and gnomAD African ancestry sample, but just 4.1% in the AA-WGS cases. The other three are more likely risk variants since they are slightly elevated in the AA-WGS cases (but also in the gnomAD African ancestry sample). A further rare variant in *PTGER4*, the gene with the highest common variant effect specifically in African Americans [22], may also be a risk allele in African ancestry individuals alone since it is also absent from Europeans, though it too is also slightly higher frequency in gnomAD.

**Table 2:** Variants in Sazonovs et al. loci in African Americans, with EP-scores within pathogenic range (EP < 0.0022)

**Gene**	**varID**	**rsID**	**Variant**	**EP-score**	**AF Case**^**a**^	**AF Control**^**b**^	**AF gN-AFR**^**c**^
SDF2L1	22−21643930−G−A	rs61739341	D141N	0.001	0.018	0.016	0.017
PTAFR	1−28150202−CT	rs140866472	A274T	0.002	0.012	0.011	0.013
PTAFR	1−28150940−G−A	rs116552042	L28F	0.004	0.0093	0.0061	0.007
PTGER4	5−40692215−G−A	rs138674524	R435Q	0.002	0.00057	0.0003	0.0008
SLC39A8	4−102263082−G−A	rs112519623	L449F	0.0008	0.0051	0.0015	0.003
IL10RA	11−117988495−C−G	rs4252250	L61V	0.005	0.041	0.044	0.045

^a^MAF in African American WGS cases

^b^MAF in African American WGS controls

^c^MAF in gnomAD African American database

### Variance explained by established rare causal variants for Crohn’s disease in African Americans

Despite the five-fold reduction in MAFs between European and African IBD and control cohorts, evaluation of allelic effect sizes is necessary to establish whether they also explain a considerably lower proportion of the risk of Crohn’s disease in African Americans. Power to establish whether a variant is a risk factor or not is low as there were only 1774 AA cases and accordingly only R161H in *SDF2L1* was even nominally significant (*p* = 0.005) in AA, yet all 8 risk alleles with European MAF > 0.01 are concordant in their observed direction of effect in AA and EA (contrast the heights of the dark and lighter-blue shades for AA, or dark and lighter reds for the two EA cohorts in Fig. 1). Furthermore, the computed odds ratios are slightly higher for half of the variants as documented in Table 1. Regarding the protective alleles, 3 of 4 with measurable effects are also nominally protective in African Americans, the exception being P1104A in *TYK2*.

Table 1 shows the variance explained in our IBD-GC AA WGS cohort and the Sazonovs et al. cohorts [10], computed as 2*pq*(lnOR)^2^ where *p* is the MAF and* q* = 1-*p*. Excluding the 11 very- or ultra-rare variants for which odds ratio computation is unreliable, averaged across the other 5 risk alleles not including *NOD2*, the rare coding variants discovered in Europeans are predicted by this method to have similar population attributable risk in African Americans as in Europeans, cumulatively 1.39%. However, more than half of this is due to R161H in *SDF2L1*, which may have an inflated estimate due to sampling variance, while the variants in *HGFAC* and *SLC93A8* also make appreciable contributions. The three established major risk factors at *NOD2* by contrast explain just 0.75% of the variance in AA, compared with up to 12% in EA. Regarding the protective variants, the lower allele frequencies result in much less protection in AA, an estimated 1% versus 7.6% in EA, although most of the latter is due to the relatively high frequency of R381Q in *IL23R* in EA.

An alternative mode of measuring the burden of rare variants is to calculate the difference between the observed number of cases and that expected if the odds ratio of each variant were 1. Cumulatively, the 19 risk variants were observed in 405 of the 1744 cases, and 318 of the 1644 controls in the AA WGS dataset, for a combined odds ratio of 1.26. For comparison, the mean odds ratio in Europeans, excluding *NOD2*, was 1.41. If we assume for the sake of direct comparison a prevalence of 1% in both African and European ancestry populations, this implies 41 excess cases per 100,000. This estimate includes the very rare variants, but since their contribution is small, restricting the analysis to the 8 rare variants above also yields a similar reduction in burden in African Americans as compared to European ancestry. Not accounting for co-occurrence of multiple variants in some individuals and summing the individual excess burden results in 59 cases per 100,000 in AA and 271 in EA from Table 1. Correspondingly, we estimate 15 versus 111 fewer cases in AA and EA respectively, due to the 6 documented protective rare variants. Cumulatively, then, the 25 rare Sazonovs et al. variants are expected to contribute to the occurrence of 44 excess cases of Crohn’s disease per 100,000 African Americans, four-fold fewer than the 160 excess cases (1 in 625) in European ancestry individuals. This mode of analysis thus agrees with the interpretation based on percent variance explained.

### Most of the known European-discovered rare variant burden for Crohn’s Disease in African Americans is due to admixture

To determine whether the lower allele frequencies in African ancestry individuals may reflect lower nucleotide diversity at these IBD risk loci in general, we computed coding region nucleotide diversity (*π*) on African and European-derived chromosomes (see Methods; Additional file 1: Fig. S1). There was no consistent pattern, with four loci showing elevated diversity and three reduced diversity on African-derived haplotypes and three with similar measures. Similar results were observed for the four loci with rare protective variants. Since genome-wide diversity is known to be ~30% greater in African than European populations, most of these genes have higher than expected diversity in Europeans relative to Africans [14], possibly indicating reduced selection outside Africa.

The Sazonovs et al. variants allele frequencies were evaluated in West Africans from the 1000 Genomes database; however, because their AFs were nearly 0, we did not include these estimates in Fig. 1. All 8 of the rare risk alleles are at a lower frequency in West Africans in the 1000 Genomes database than in our AA WGS cohort study. At *NOD2*, the R702W MAF is close to 1% in both West Africans and the gnomAD AA cohorts, whereas G908R and 1007fs are absent. Four of the other 5 variants are also absent or nearly so in West Africans in the 1000 Genomes database, the exception being rs16844401 in *HGFAC*, which has a MAF of 2.3%, compared with 6.5% in non-Finnish Europeans. Just one of the protective alleles, rs41267765 in *TAGAP*, has a MAF greater than 1% in Africans, and it is the only variant in the dataset predicted [36] to pre-date human origins.

**Fig. 2:**
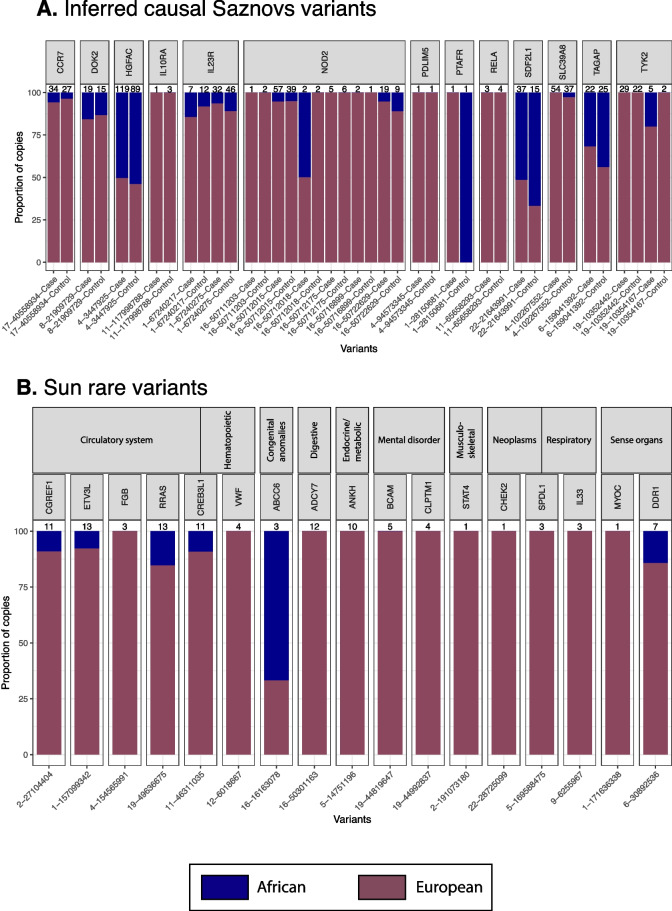
Admixture proportion of rare IBD variants. **A** Proportion of copies from African (blue) and European (brown) haplotypes containing Sazonovs et al. [10] inferred causal variants for carriers in the African American WGS cohort. **B** Proportion of copies from African and European haplotypes containing Sun et al. rare variants [24] associated with different disease groups for carriers in the African American WGS cohort. The numbers above each bar represent the total number of copies in both **A** and **B**

These results suggest that the presence of most of the Sazonovs et al. variants in African Americans may be predominantly due to admixture. To confirm this, we used Gnomix [31] to paint each of the chromosomes on which the genes are found, allowing inference of the genetic background of origin for each variant in each individual. Strikingly, as seen in Fig. 2A, at *CCR7*, *DOK2*, *SLC39A8*, and *NOD2*, just 17/300 risk alleles (less than 5%) appear to be on African haplotypes. In addition, all 14 of the 15 very or ultra-rare variant instances at four genes (*RELA*, *PTAFR*, *PDLIM5*, and *IL10RA*) are European-derived. At *HGFAC* and *SDF2L1*, the two loci with common risk variants, the proportions of African and European-derived risk variants are close to 50%, which is still well below the genome-wide average of non-pathogenic alleles. The protective variants at *IL23R*, *TYK2*, and *TAGAP* are also mostly European-derived, to varying degrees. As a control for these inferences, we show in Additional file 1: Fig. S2 that the major alleles follow the expected distribution for African Americans of ~80% African ancestry, in both cases and controls, reflecting the known contribution of admixture to African ancestry genetic proportions.

**Fig. 3:**
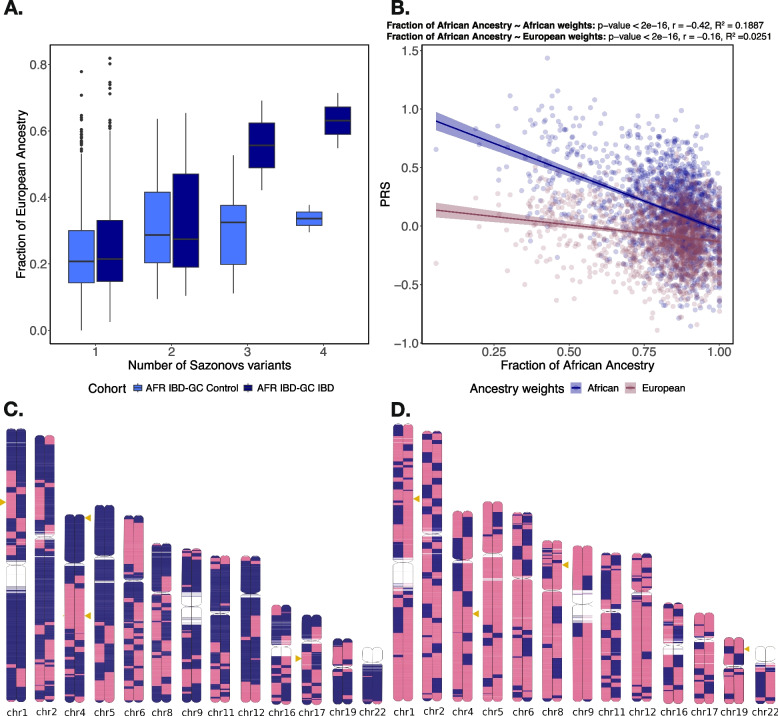
Relationships between admixture proportion and genetic risk. **A** Genome-wide fraction of European ancestry per individual against total number of Sazonovs et al. variants carried per individual. **B** PRS derived from African weights (blue) and European weights (red) vs. fraction of African ancestry. **C** Chromosome painting for an African American control carrying four inferred causal Sazonovs et al. variants. **D** Chromosome painting for an African American IBD case carrying four inferred causal Sazonovs et al. variants. The blue and pink colors in the chromosome paintings represent African and European, respectively

A corollary of these results is that there is a highly significant correlation between the proportion of the genome, at the 13 chromosomes, derived from European ancestry, and risk of CD. Figure 3A shows that for the 19 causal risk variants, fraction of European ancestry increases with number of causal rare variants (*p* = 1.2 × 10^–9^), slightly more in the cases than controls. The ideograms in Fig. 3 panels C and D show admixture segments by chromosome for a control and case individual respectively, each with 4 causal variants (gold arrowheads) where the European ancestry (pink intervals) is clearly greater in the case. Gnomix [31] overestimates the number of breakpoints due to error in phasing, but this should not affect the ancestry inference at most loci.

We also asked whether African ancestry proportion correlates with polygenic risk score for the common variant risk for IBD. Using a PRS generated using liability-scale allelic effect weights computed in the UK Biobank for 215 established common risk variants, as reported in [22], a weak but highly significant negative correlation (*r* =  − 0.16, *p* < 10^–16^) between African ancestry proportion and PRS was observed for the AA cases in the IBD-GC WGS dataset. This appears to be because there is a slight excess of risk variants that are more common in Europeans, so admixture tends to increase the proportion of risk variants. Substituting effect weights estimated for African Americans markedly increased the correlation (*r* = -0.42) as seen in Fig. 3B. This is likely due to 55% of the risk variants having larger effect sizes observed in AA, increasing the contribution of admixed variants to the PRS. It also suggests that the slightly elevated prevalence of cases in the top percentiles of risk in AA with this AA-weighted PRS [22] is actually due to increased admixture, noting a small but significant correlation between proportion of European ancestry and IBD prevalence in the IBD-GC cohort. Presumably, there are also common variants yet to be discovered in poorly sampled African ancestry populations that would produce opposing effects on overall risk but are not captured by the existing PRS.

### Similar biases are observed for other common complex diseases

Sun et al. [24] reported 975 coding-wide significant variants for a wide range of diseases in the initial release of the UK Biobank WES data supplemented with FinnGen WES. We evaluated the relative burden in the gnomAD African and NFE cohort subsets using the same approach as for the IBD risk loci, but without differentiating between cases and controls. Across all loci, there was a highly significant difference in frequency of pathogenic rare variants (MAF < 0.05), with an average MAF of 0.003 in AA and 0.017% in NFE (*t*-test, *p* = 2 × 10^–16^). Figure 2B shows that just 8 of 105 variants detected in the IBD-GC WGS cohort were observed on African-derived haplotypes, consistent with the majority being present due to admixture. These are partitioned by disease category in Fig. 2B, and reduction in rare variant allele frequency was significant for the loci associated with diseases of the circulatory system, sense organs, and endocrine/metabolic system, and trending for congenital abnormalities, digestive, genitourinary, hematopoietic, musculoskeletal, mental, and respiratory disorders. We emphasize that in each case, the evaluated variants were discovered by association studies carried out in a very large European ancestry cohort study and that comparative discovery data for AA populations is not yet available.

## Discussion

The major finding of this study is that rare coding variants discovered by WES in large European-ancestry cohort studies overwhelmingly contribute to disease risk in African Americans as a result of admixture, in whom they consequently explain between just one-fifth and one quarter as much variance as in non-Hispanic Whites (Table 1). This result extends observations we made a decade ago [16] pertaining to the major *NOD2* risk variants for Crohn’s disease, to a dozen other IBD loci [10], and more generally to 23 other loci influencing ten classes of congenital disease [24]. The risk reduction agrees with expectations given that the proportion of the genome derived from European ancestry introgression in African Americans is typically in the range of 20–25% [37, 38], on the supposition that almost all the rare alleles derive from the European-derived haplotype, as confirmed by chromosome painting.

The result should not be interpreted as implying that disease incidence would be proportionately lower in African Americans in the absence of admixture. For one thing, the rare variant contribution to disease is a minor fraction of the genetic component, and the heritability of IBD is less than 50%. In fact, epidemiological data suggests that prevalence of IBD is approaching 1% in all ancestry groups in the US [39] and has risen to that level rapidly over the course of the past century as a result of yet-to-be clarified behavioral and environmental factors associated with modernization. For another, rare variant effects that have arisen in Europeans are likely paralleled by population-specific rare variants that will be discovered in Africans or Asians once appropriately powered association studies are performed in diverse populations. Indeed, five candidate variants in Table 2 with low EP [27] but frequencies between 1 and 5% in the AA-WGS cohort, and absent in Europeans, were seen just in the dozen IBD risk genes implicated by Sazonovs et al. [10]. The rare variant effects documented here are simply those discovered in Europeans. Furthermore, it should be appreciated that admixture generally has the beneficial effect of masking deleterious effects of population-specific rare variants by reducing the probability of homozygosity [40].

It can be estimated from Table 1 that approximately one in 625 European-ancestry Americans have IBD that would not otherwise occur in the absence of the 25 rare variants surveyed here. Extraordinarily, almost two-thirds of this risk is attributable to a handful of variants in the *NOD2* gene, which does not appear to contribute to IBD risk in Africans. All three of the established variants with minor allele frequencies up to 5% and odds ratios greater than 2 [41], as well as several ultrarare variants with even larger ORs, are estimated to be younger than 1000 generations [36] and are likely to have arisen in Europe. Nucleotide diversity in *NOD2* is significantly lower in Africans than Europeans (Additional file 1: Fig. S1), as is tolerance of loss-of-function mutations (Table 3), both implying a change in selection pressure as humans migrated into Europe such that the derived alleles now associated with Crohn’s disease may have been positively selected. It seems unlikely that selection against homozygotes due to Crohn’s disease has prevented the variants from becoming common, since the disease is thought to have been very rare until a few generations ago. Weak soft selection favoring variants that would have been deleterious in carriers throughout most of human history appears more likely, but if immune-related then the causal pathogen is unknown. Although risk alleles at *NOD2* have been postulated to have offered protection against the Black Plague [42], this is doubtful since ancient DNA does not show evidence for changes occurring at the gene in Europe in the Middle Ages [43].

**Table 3:** Mutability statistics for IBD loci

**Gene**	**Age**^**a**^	**EU RVIS**^**b**^	**AA RVIS**^**b**^	**pLI**^**c**^	**LOEUF**^**d**^	**pi**_**AA**_**/pi**_**EU**_	**pi**_**AA**_
**Genes with elevated nucleotide diversity in Europeans**							
NOD2	679	1.88	-0.32	0	1.36	0.38	0.0003
DOK2	599	0.15	-0.40	0	1.07	0.53	0.0003
SLC93A8	1283	0.58	-0.25	0.67	0.47	0.56	0.0003
**Genes with reduced nucleotide diversity in Europeans**							
CCR7	1029	-0.35	-0.44	0.06	0.78	4.08	0.0004
SDF2L1	690	n/a	n/a	0.19	0.95	4.73	0.0003
RELA	651	-0.61	-0.49	1	0.18	11.0	0.0001
**Genes relatively tolerant of mutations**							
PTAFR	1508	-0.15	0.38	0.24	0.85	12.78	0.0002
HGFAC	1507	1.16	-0.09	0	0.87	1.06	0.0011
PDLIM5	480	1.68	0.82	0	0.55	0.83	0.0007
IL10RA	219	1.47	0.48	0.02	0.69	0.90	0.0009
**Genes harboring protective rare variants**							
IL23R	2830	1.61	0.35	0	0.61	1.09	0.0005
TYK2	933	0.13	-0.78	0	0.56	0.69	0.0002
TAGAP	27,489	0.02	0.19	0.85	0.42	2.46	0.0001
CARD9	532	-0.58	-0.71	0	1.13	0.98	0.0007

^a^From Human.Genome.Dating database, TMRCA point estimate in generation time for most common causal variant (first listed) in Table 1, using TGP datasource. 1000 generations correspond to 20,000–25,000 years

^b^From RVIS database

^c^From pLI database, inferred from European ancestry genomes

^d^LOEUF score European estimates

Other demographic processes appear to have influenced polymorphism at the implicated genes, but no clear patterns relating the rare risk variants to genic mutability were seen. We recorded four measures of tolerance to mutations, pLI, LOEUF, and RVIS in EA and AA (Table 3) and also compared these values with the standard population genetic parameter of nucleotide diversity, *π*. This analysis revealed a group of genes with clearly reduced nucleotide diversity in Europeans (*CCR7*, *SDF2L1*, *RELA*, *PTAFR*), one with elevated nucleotide diversity in Europeans (*NOD2*, *DOK2*, *SLC93A8*), and a group relatively tolerant of mutations (*HGFAC*, *PDLIM5*, *IL10RA*). The four genes harboring protective rare variants also span the full range of mutability. The most conspicuous observation concerning these genes is the relatively old age of rs41267765 in TAGAP (27,850 generations), which must have arisen in Africa, yet two-thirds of the rare variants appear on European-derived chromosomal segments. Notably, the two risk variants that appear to be equally likely to be African- or European-derived, in *HGFAC* and *SDF2L1*, are also present in Yorubans in the 1000 Genomes database [14] at low frequency. Combined with their young age, this suggests introgression into the African gene pool prior to the initiation of the slave trade that gave rise to admixture at most of the loci considered in this study [44, 45].

A limitation of this study is that power to associate rare variants with disease remains low and more genes will surely be identified as sequence based GWAS increase in size and diversity. Measures of evolutionary conservation, notably the EP-score which measures the probability of observing a specific allele in humans given phylogenetic diversity [27], are strongly enriched for pathogenicity, but not sufficient to identify causal variants. Given that rare alleles tend to be specific to the major human ancestry groups [4, 13] and given the sparsity of “goldilocks” variants detected in Biobank studies, it appears that the genes harboring rare variants of relatively large effect are likely to differ among populations.

## Conclusions

Loss of function protective variants are of such interest as pharmaceutical targets, since perturbation is less likely to be harmful, that expanded exome and genome sequencing of disease cases is most likely to be informative if it is inclusive of the full range of human diversity. More generally, whether considering rare variant burden or polygenic risk assessment, our results illustrate the importance of considering admixture when performing cross-ancestry genetic evaluation.

### Supplementary Information

**Additional file 1: Fig. S1.** Nucleotide diversity estimates for the CDS regions. **Fig. S2.** Ancestral origins of gene copies.

## Data Availability

The gnomAD V3.1.2 (https://gnomad.broadinstitute.org/downloads#v3) population-specific AF data was accessed using the Ensemble Variant Effect Predictor (VEP) (https://useast.ensembl.org/info/docs/tools/vep/index.html). The IBD/control African American WGS data used in this study are available at dbGaP: phs001642.v1.p1 (https://www.ncbi.nlm.nih.gov/projects/gap/cgi-bin/study.cgi?study_id=phs001642.v1.p1). EP-score annotations were received from the mypeg resource (http://mypeg.info/ep). http://ftp.1000genomes.ebi.ac.uk/vol1/ftp/data_collections/1000G_2504_high_coverage/working/phase3_liftover_nygc_dir European and African WGS reference samples obtained from Phase 3 of 1000 Genomes project used for global and local ancestry inference. The Homo Sapien refseq genomic annotation file used to assess genomic coordinates of CDS regions can be found here: https://ftp.ncbi.nlm.nih.gov/refseq/H_sapiens/annotation/GRCh38_latest/refseq_identifiers/GRCh38_latest_genomic.gff.gz. The pLI and LOEUF score annotations were extracted from https://hgdownload.soe.ucsc.edu/gbdb/hg38/gnomAD/pLI/pliByGene.bb. The RVIS annotations were extracted from: https://genic-intolerance.org/data/GenicIntolerance_v3_12Mar16.txt. Relevant code corresponding to the analyses can be accessed via the following GitHub repository: https://github.com/courtneyastore/rarevariant_admixture.
