# Supplementary material for: The role of admixture in the rare variant contribution to inflammatory bowel disease
Source: Genome Med. 2023 Nov 15;15:97. doi: 10.1186/s13073-023-01244-w (PMC10647102; doi:10.1186/s13073-023-01244-w)
Supplement: Supplementary file 1 — Additional file 1: Fig. S1. Nucleotide diversity estimates for the CDS regions. Fig. S2. Ancestral origins of gene copies. [file 13073_2023_1244_MOESM1_ESM.docx]

**Additional File 1**

**
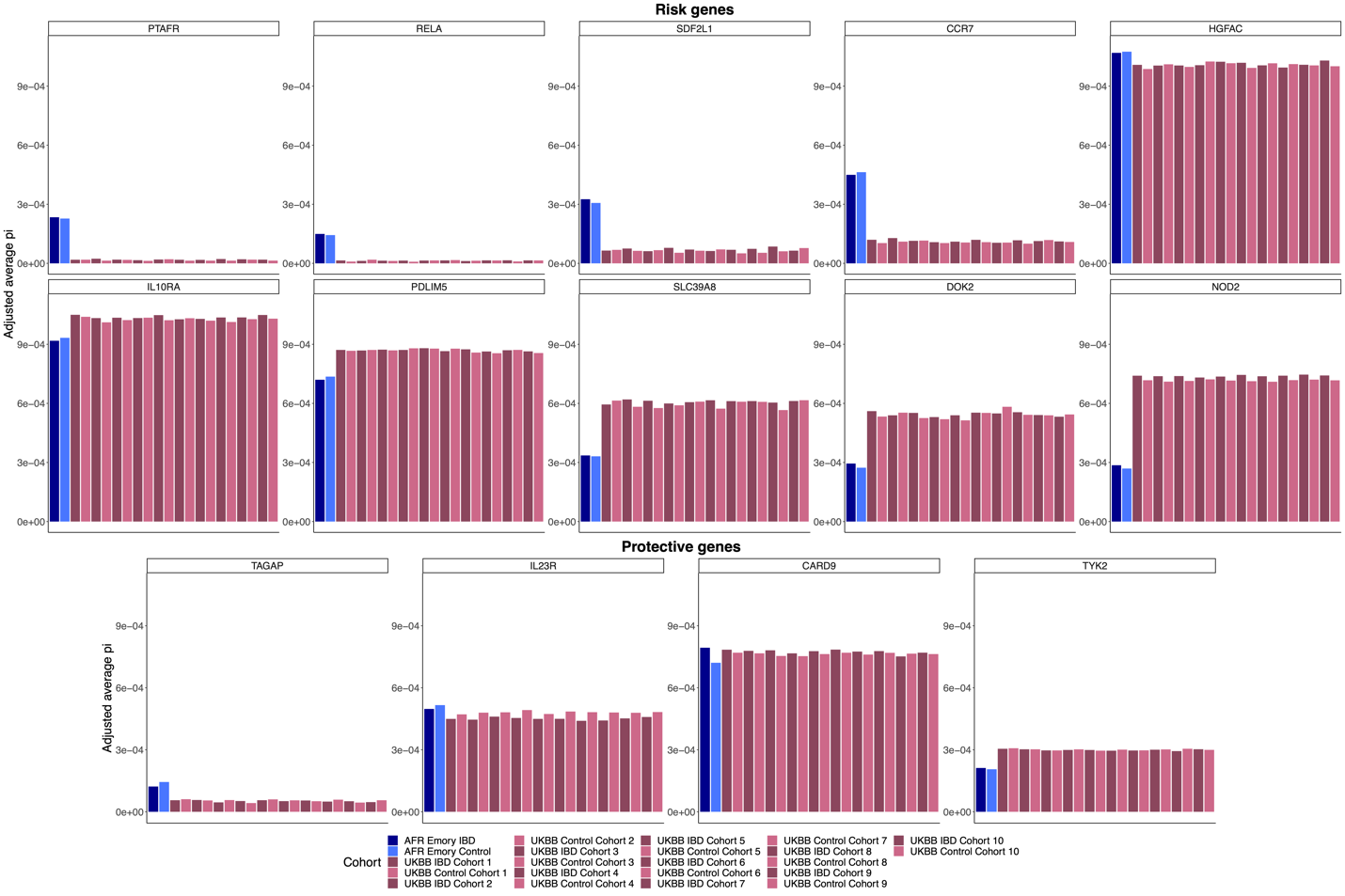
**

**Figure S1:** Nucleotide diversity estimates for the CDS regions of the Sazonovs et.al. IBD loci for African American case/control and 10 down-sampled UKBB European case/control cohorts.


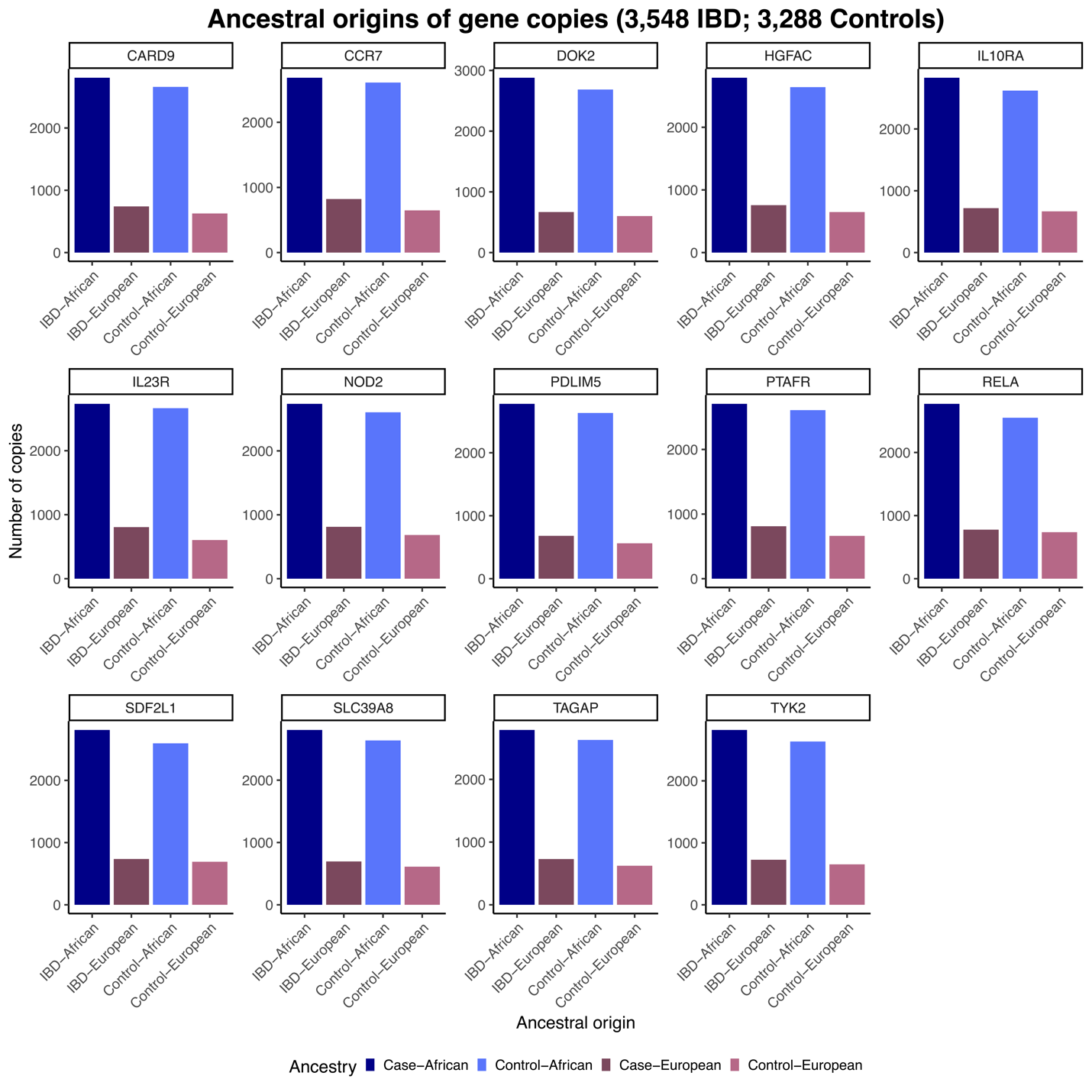


**Figure S2:** Number of copies from African and European for haplotype regions of Sazonovs et.al. inferred causal variants for African American individuals irrespective of their variant carrier status.
